# Combination of phage therapy and cefiderocol to successfully treat *Pseudomonas aeruginosa* cranial osteomyelitis

**DOI:** 10.1093/jacamr/dlac046

**Published:** 2022-05-05

**Authors:** Patricia J. Simner, Jerald Cherian, Gina A. Suh, Yehudit Bergman, Stephan Beisken, Joseph Fackler, Martin Lee, Robert J. Hopkins, Pranita D. Tamma

**Affiliations:** 1 Johns Hopkins University School of Medicine, Baltimore, MD, USA; 2 The Mayo Clinic, Rochester, MN, USA; 3 Ares Genetics, Vienna, Austria; 4 Adaptive Phage Therapeutics, Gaithersburg, MD, USA

## Abstract

**Background:**

*Pseudomonas aeruginosa* has the ability to exhibit resistance to a broad range of antibiotics, highlighting the importance of identifying alternative or adjunctive treatment options, such as phages.

**Patients and methods:**

We report the case of a 25-year-old male who experienced an accidental electrocution resulting in exposed calvarium in the left parieto-temporal region, complicated by a difficult-to-treat *P. aeruginosa* (DTR-*P. aeruginosa*) infection. Cefiderocol was the sole antibiotic with consistent activity against six bacterial isolates obtained from the infected region over a 38 day period.

**Results:**

WGS analysis identified a *bla*_GES-1_ gene as well as the MDR efflux pumps MexD and MexX in all six of the patient’s ST235 DTR-*P. aeruginosa* isolates, when compared with the reference genome *P. aeruginosa* PA01 and a *P. aeruginosa* ST235 isolate from an unrelated patient. After debridement of infected scalp and bone, the patient received approximately 6 weeks of cefiderocol in conjunction with IV phage Pa14NPøPASA16. Some improvement was observed after the initiation of cefiderocol; however, sustained local site improvement and haemodynamic stability were not achieved until phage was administered. No medication-related toxicities were observed. The patient remains infection free more than 12 months after completion of therapy.

**Conclusions:**

This report adds to the growing literature that phage therapy may be a safe and effective approach to augment antibiotic therapy for patients infected with drug-resistant pathogens. Furthermore, it highlights the importance of the GES β-lactamase family in contributing to inactivation of a broad range of β-lactam antibiotics in *P. aeruginosa*, including ceftolozane/tazobactam, ceftazidime/avibactam and imipenem/relebactam.

## Introduction

*Pseudomonas aeruginosa* is a ubiquitous organism that remains an important nosocomial pathogen.^1^ It has an impressive capacity to exhibit resistance to all available antibiotics through the development of mutations in or induction of chromosomal genes, in conjunction with the emergence and dissemination of transferable resistance determinants.^1^ Four β-lactam agents (i.e. ceftolozane/tazobactam, ceftazidime/avibactam, imipenem/cilastatin/relebactam, and cefiderocol) with activity against *P. aeruginosa* with difficult-to-treat resistance (DTR-*P. aeruginosa*) have received approval by the US FDA.^2^ Unfortunately, acquisition of resistance to all of these agents by *P. aeruginosa* has been encountered, leading to a renewed interest in phage therapy. Lytic phages have been used therapeutically for the last century for their ability to infect and lyse bacteria for the treatment of bacterial infections.^3^ We report a case of a patient with DTR-*P. aeruginosa* cranial osteomyelitis that was successfully treated with cefiderocol in conjunction with phage therapy.

## Methods and results

### Clinical case

**Figure 1.**
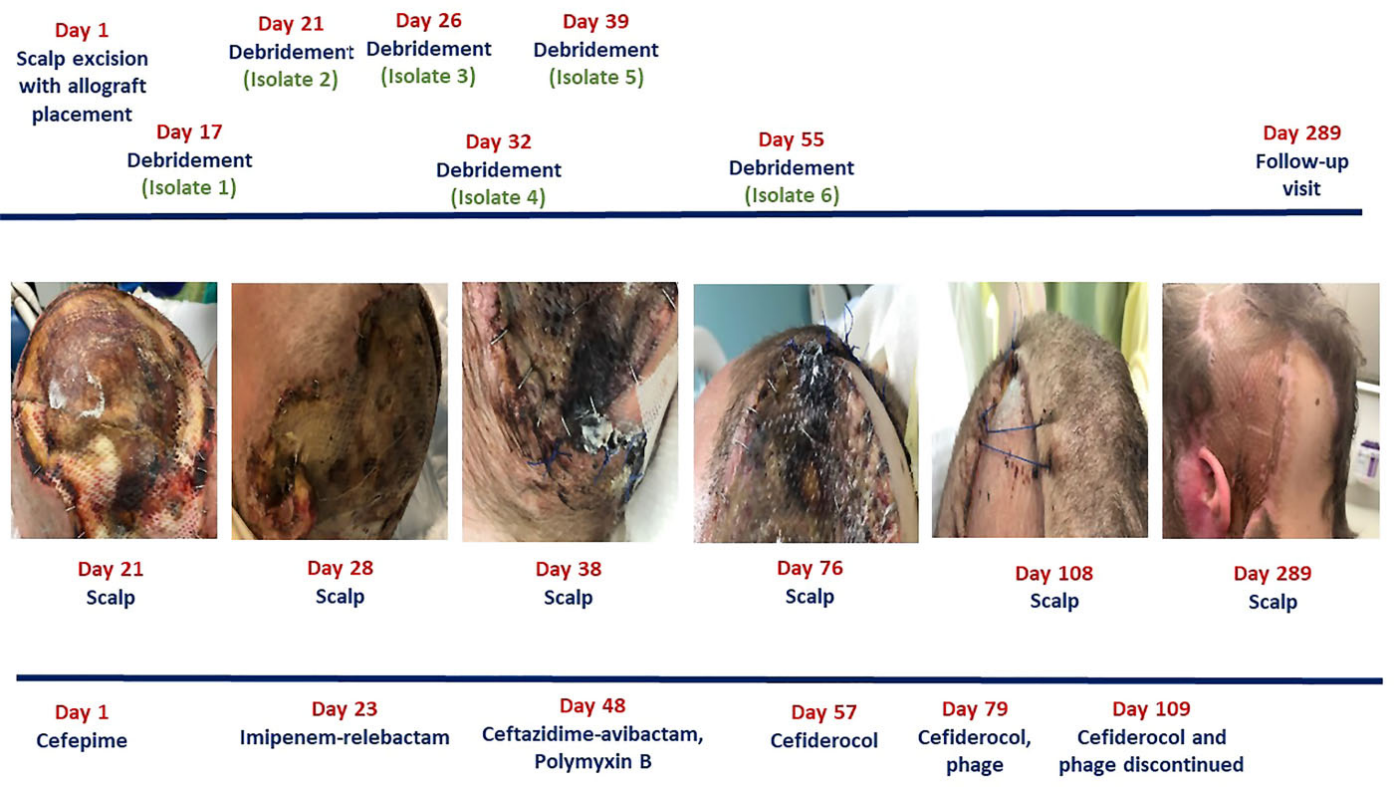
General overview of clinical course. Cultures without recovery of *P. aeruginosa* not included. Only antibiotic agents with Gram-negative coverage included for simplicity.

A 25-year-old male with no significant past medical history presented to medical care after an accidental electrocution (Figure 1). He experienced 22% total body surface area third- and fourth-degree burns. Burn wounds of the skull were complicated by a soft tissue defect resulting in exposed calvarium in the left parieto-temporal region. The patient underwent excision of the scalp wound with surgical debridement of the skull, allograft placement and partial closure of the defect with a musculocutaneous flap. On Day 16, the patient developed a temperature to 39.8°C and haemodynamic instability. A fluid collection was identified under his flap. A WBC count and C-reactive protein (CRP) measurement obtained on Day 16 were 42 600 cells/mL and 12.8 mg/dL, respectively. The collection was drained on Day 17 and DTR-*P. aeruginosa* was recovered. Additional debridement occurred on Day 21 with recovery of DTR-*P. aeruginosa* once again. The patient was transitioned to imipenem/cilastatin/relebactam on Day 23 (Table 1). He underwent additional skull debridement on Day 26 and Day 32. Cultures from both procedures recovered DTR-*P. aeruginosa* isolates exhibiting non-susceptibility to imipenem/relebactam, prompting a transition to ceftazidime/avibactam and polymyxin B on Day 48.

**Table 1.** AST results using BMD for six *P. aeruginosa* isolates recovered from the same patient over a 38 day period

Isolate number	Day of culture	MIC (mg/L)															
		AMK	ATM	CAZ	CZA	CIP	CST	C/T	FDC	FEP	GEN	IPM	IMR	MEM	PLZ	TOB	TZP
Isolate 1	Day 17	>32	16	>16	>32	>2	≤1	>8	0.5	>16	>8	16	2	>8	>4	>8	32
Isolate 2	Day 21	>32	>16	>16	>32	>2	≤1	>8	0.5	>16	>8	16	2	>8	>4	>8	32
Isolate 3	Day 26	>32	>16	>16	32	>2	≤1	>8	0.5	>16	>8	16	2	>8	>4	>8	32
Isolate 4	Day 32	>32	>16	>16	32	>2	≤1	>8	0.5	>16	>8	16	4	>8	>4	>8	>64
Isolate 5	Day 39	>32	>16	>16	32	>2	≤1	>8	0.5	>16	>8	16	4	>8	>4	>8	>64
Isolate 6	Day 55	>32	>16	>16	4	>2	≤1	>8	0.5	>16	>8	16	4	>8	>4	>8	>64

The reduction in MIC of CZA for isolate 6 was confirmed by repeat testing.

AMK, amikacin; ATM, aztreonam; CAZ, ceftazidime; CZA, ceftazidime/avibactam; CIP, ciprofloxacin; CST, colistin; C/T, ceftolozane/tazobactam; FDC, cefiderocol; FEP, cefepime; GEN, gentamicin; IPM, imipenem; IMR, imipenem/relebactam; MEM, meropenem; PLZ, plazomicin; TOB, tobramycin; TZP, piperacillin/tazobactam.

Although cefiderocol was the only remaining β-lactam agent exhibiting consistent activity against all six isolates (Table 1), in an effort to preserve its activity, a decision was made to withhold its administration until surgical interventions were exhausted. After the patient underwent full thickness craniectomy on Day 55, he was transitioned to cefiderocol on Day 57 and phage therapy was requested. Between Days 57 and 78 the affected region of the skull showed some improvement; however, the patient continued to have episodes of temperature and haemodynamic stability.

Three of the six *P. aeruginosa* isolates were shipped to Adaptive Phage Therapeutics (APT) to identify suitable phage matches. The isolates were susceptible to a lytic *P. aeruginosa* phage, Pa14NPøPASA16, originally identified in Israel. A mono-phage preparation with Pa14NPøPASA16, manufactured under cGMP conditions (Supplementary Methods, available as Supplementary data at *JAC-AMR* Online), was initiated on Day 79 at a concentration of 1.7 × 10^11^ PFU diluted in 25 mL of normal saline (NS) and administered IV every 12 h as a 30 min infusion. NS bags containing the phage were thoroughly mixed before each infusion. Tubing was flushed with at least 25 mL of NS after each infusion. Phage infusions were spaced at least 3 h apart from the cefiderocol, to theoretically enable phage to proliferate in sufficient quantities of host before lysis.

Within 24 h of the first dose of phage therapy the CRP increased from 4.6 mg/dL to 6.4 mg/dL and the WBC count increased from 6580 to 14 800 cells/mL. Within 48 h of administration of the first dose of phage therapy, both inflammatory markers normalized. The patient demonstrated local wound improvement with no further episodes of temperature or haemodynamic instability. No additional DTR-*P. aeruginosa* isolates were recovered. During receipt of phage therapy, weekly inflammatory markers, electrolytes, liver function tests and complete blood counts were obtained. No abnormal laboratory values or findings on clinical exam suggestive of medication toxicities were observed. Antibiotic and phage therapy were discontinued on Day 109 and the patient was discharged from the hospital shortly after. The patient’s left scalp region showed significant improvement at follow-up visits 6 months and 12 months after the discontinuation of therapy (Figure 1). The patient remains infection free more than 12 months after completion of anti-infective therapy.

### Antimicrobial susceptibility testing (AST)

Bacterial genus and species were identified using MALDI TOF (Bruker Daltonics Inc.). Initial AST results were determined using the BD Phoenix Automated System (BD Diagnostics). After resistance to traditional β-lactam agents was identified, AST for novel β-lactams was determined using lyophilized Sensititre broth microdilution (BMD) GN7F and MDRGN2F BMD panels (Thermo Fisher Scientific). CLSI interpretive criteria were applied to determine *P. aeruginosa* susceptibility.^4^ Isolates were stored at −80°C in Microbank tubes (Pro-Lab Diagnostics) until further testing was performed.

### Phage susceptibility testing

APT evaluated their existing phage library to identify a phage with activity against three clinical isolates provided by the patient. The library consists of a collection of well characterized phages isolated from environmental samples and identified as pure, free of deleterious genes or elements of lysogeny, and capable of lysing pathogenic bacteria. APT performed phage susceptibility testing by evaluating the bacteria’s ability to establish a logarithmic growth phase in the presence of phage compared with an uninfected bacterial control (Supplementary Methods). Pa14NPøPASA16 inhibited growth of the patient’s isolates for at least 20 h longer compared with a control tested in the absence of the phage. The prolonged duration of growth inhibition suggests that Pa14NPøPASA16 had independent activity against the patient’s *P. aeruginosa* isolates.

### WGS

WGS was conducted to understand the mechanisms contributing to the DTR-*P. aeruginosa* phenotype. Frozen isolates were subcultured twice to tryptic soy agar with 5% sheep blood. Genomic DNA was extracted from the six isolates using the DNeasy PowerSoil Pro Kit (QIAGEN Inc.). WGS was conducted using Illumina MiSeq short-read sequencing (Illumina) and bioinformatics analysis was performed by Ares Genetics, both as previously described.^5^ All six *P. aeruginosa* isolates belonged to multilocus ST235.

Multiple sequence alignment of the six isolates were compared to both the reference genome *P. aeruginosa* PA01 as well as to a clinical isolate of *P. aeruginosa* ST235 from an unrelated patient. The ‘reference’ clinical *P. aeruginosa* ST235 isolate was obtained from an 81-year-old male with a complicated urinary tract infection at The Johns Hopkins Hospital in December 2016. AST of the reference ST235 isolate revealed resistance to all traditional β-lactam agents. The reference ST235 isolate exhibited susceptibility to all the novel β-lactams: cefiderocol (2 mg/L), ceftazidime/avibactam (4 mg/L), ceftolozane/tazobactam (1/4 mg/L) and imipenem/relebactam (2/4 mg/L).

Compared with the PA01 isolate, the patient’s isolates contained a *bla*_OXA-488_ (member of the *bla*_OXA-50_ family), *bla*_PDC-35_ and *bla*_GES-1_. Several protein mutations were identified in AmpR, AmpD, AmpC and OprD. MexD and MexX efflux pumps were identified in the patient’s isolate; no full-length close homologies of these proteins were identified in the reference PA01 isolate. Compared with the ‘reference’ ST235 isolate, the only additional β-lactamase gene identified was *bla*_GES-1_. No differences in AmpR, AmpD, AmpC or OprD sequences were identified. No close homologues of MexD or MexX identified in the clinical isolates were identified in the reference ST235 isolate.

## Discussion

We report the case of an unfortunate man who was previously healthy and sustained an electrocution-associated exit wound resulting in extensive injury to his left parieto-temporal skull and scalp. His course was complicated by the development of a skull osteomyelitis due to DTR-*P. aeruginosa*. WGS revealed elevated MICs of all clinically available β-lactam lactams, except cefiderocol, which were likely due to the presence of *bla*_GES-1_, a β-lactamase gene, in combination with the presence of the MDR efflux pumps MexD and MexX,^6^ as this combination of resistance markers was present in the patient’s isolates but not in the PA01 reference genome or the reference clinical ST235 isolate.

The *bla*_GES_ family are Class A β-lactamase genes generally encoded on class 1 integrons—increasing their potential for widespread dissemination, especially when found in high-risk clones such as ST235.^7,8^ Although GES enzymes have traditionally been considered ESBLs, point mutations can expand their activity to include carbapenem substrate utilization (e.g. GES-5, GES-6).^9^ Several reports have demonstrated that *P. aeruginosa* producing GES enzymes are capable of exhibiting resistance to ceftolozane/tazobactam,^9–11^ ceftazidime/avibactam^10,11^ and imipenem/relebactam,^12–15^ similar to the current case. The MexD and MexX MDR efflux pumps identified in our patient’s isolates likely provided a furthering of β-lactam resistance, as they have been previously associated with the extrusion of β-lactam antibiotics.^6^ Although less common in the USA, in several regions of the world, GES enzymes are becoming an important contributor to DTR-*P. aeruginosa* phenotypes.^7,8,16,17^ Our patient had no history of international travel and was otherwise healthy prior to his current hospitalization suggesting likely nosocomial acquisition.

Despite the heavy burden of disease with a DTR-*P. aeruginosa* isolate, our patient achieved clinical cure of his infection, and in the absence of any treatment-related adverse events. The lack of toxicities in this case is consistent with the consistent safety record of therapeutic phages as reported in the literature.^3^ Marked improvement in the appearance of the wound and normalization of vital signs occurred only after the initiation of phage therapy and was less apparent during the interim period when cefiderocol therapy was administered alone. Nevertheless, it is unknown whether improvement would have eventually occurred without the addition of phage therapy. The vast majority of clinical experiences generated over the past two decades have evaluated the role of phages in conjunction with, rather than in place of, antibiotic therapy providing limited insight into the incremental benefit of phages in contributing to clinical cure.^3^ We postulate, however, that there may be a synergistic effect of the phage-antibiotic combination. Bacteria can readily downregulate receptors used by phages to penetrate bacteria and lead to phage resistance, while simultaneously reducing bacterial virulence, underscoring an advantage in using both treatments concurrently.^18,19^

As an example, to ward off the activity of ΦFG02 and ΦCO01 phages, which use the *Acinetobacter baumannii* capsule as a receptor, *A. baumannii* isolates developed loss-of-function mutations in genes responsible for capsular biosynthesis—disrupting phage entry while increasing the vulnerability of *A. baumannii* mutants to human complement.^18^ This is further exemplified by the clinical case of a 76-year-old patient with a *P. aeruginosa* aortic graft infection successfully treated with a phage-antibiotic combination that exploited a similar evolutionary trade-off. The patient was treated with phage OMKO1, which uses the MexAB/XY-OprM MDR efflux systems of *P. aeruginosa* as bacterial receptors.^19,20^ As *P. aeruginosa* isolates reduced expression of these efflux systems to inhibit OMKO1 binding, they also inadvertently reinstated the activity of antibiotics that were previously removed by efflux pumps.^19^ Knock-out experiments to identify the receptor for Pa14NPøPASA16 used in the current case were not undertaken and its receptor remains unknown.

In summary, the above case supports a growing body of literature that phage therapy may be a safe and effective approach that may have additive value when combined with antibiotic therapy for patients infected with challenging infections—including those caused by DTR bacterial isolates. Clinical trials are currently underway to define the specific subpopulations and infection types that may benefit the most from potential antibiotic-phage synergy.

## Supplementary Material

dlac046_Supplementary_Data

## Transparency declarations

P.J.S. reports grants and personal fees from Accelerate Diagnostics, OpGen Inc. and BD Diagnostics; grants from bioMérieux Inc., Affinity Biosensors and Hardy Diagnostics; and personal fees from Roche Diagnostics, Shionogi Inc. and GeneCapture, outside the submitted work. S.B. is an employee of Ares Genetics. J.F., M.L. and R.J.H. are employees of Adaptive Phage Therapeutics (APT). APT provided the phage used in this clinical case. G.A.S. reports a business relationship with APT. All other authors have nothing to disclose.

## Supplementary data

Supplementary Methods are available as Supplementary data at *JAC-AMR* Online.
