# Supplementary material for: Combination of phage therapy and cefiderocol to successfully treat Pseudomonas aeruginosa cranial osteomyelitis
Source: JAC Antimicrob Resist. 2022 May 5;4(3):dlac046. doi: 10.1093/jacamr/dlac046 (PMC9071546; doi:10.1093/jacamr/dlac046)
Supplement: dlac046_Supplementary_Data [file dlac046_supplementary_data.docx]

**Supplementary data**

**Supplementary Methods**

**Bacterial Isolate and Phage Receipt.** Adaptive Phage Therapeutics acquired *P. aeruginosa* phage Pa14NPøPASA16 and *P. aeruginosa* host Pa14 from Hadassah Medical Center in July 2019. The lytic phage sample received was plaque purified on Tryptic Soy Agar (TSA) media with a soft agar overlay containing bacterial isolate Pa14. An isolated plaque was cut from the agar and eluted in RMBio Phosphate Buffered Saline (PBS). The eluted plaque was serial diluted and then plated on TSA media with a soft agar overlay containing the Pa14 isolate. Plates were incubated at 37°C overnight.

**Plate Lysate Amplification**. The plaque purified phage was initially amplified via plate lysates in which the bacterial lawn was infected with Pa14NPøPASA16 using a soft agar overlay. The plate lysates were then incubated overnight at 37°C. Plates yielding bacteriophage lawns were eluted with RMBio PBS solution, filtered through a 0.22 μm filter.

**Small Scale Amplification**. Pa14 was grown in TSB at 37°C in a shaking incubator overnight. Using a pipette, this culture was used to seed one 200ml flask containing 100 ml of Tryptic Soy Broth (TSB) media incubated at 37°C shaking at 175 RPM. Bacterial growth was monitored every 30 minutes via spectrophotometric methods. Once mid-log phase growth of the culture was achieved (OD600~0.1), the 100 ml culture was infected with the amplified plate lysate of PASA16 at a Multiplicity of Infection (MOI) of ~0.1. Each flask was re-incubated at 37°C shaking at 175 RPM. Culture viability was monitored at 1-hour intervals for detection of lysis. Once lysis was observed, cellular debris was removed via centrifugation and 0.22 μm filtration.

**Large Scale Amplification**. Two 4 L flasks containing 1.8 L of non-animal origin TSB media was seeded with the selected producer bacterial strain and incubated at 37°C shaking. Bacterial growth was monitored via spectrophotometric methods until the growth reached an optical density between 0.08-0.1. Both large batch cultures were infected with the target bacteriophage stock from small batch amplification. The flask was re-incubated at 37°C while shaking. Culture viability was monitored visually for detection of lysis. At 2.5 hours post infection, Benzonase was added to each flask to a final concentration of 2 μg/mL per flask. After the lysis event, both flasks were pooled. The supernatant was centrifuged and sequentially filtered through 0.88 μm, 0.45 μm, and 0.22 μm filters.

**Concentration, Purification, and Aseptic Filling.** Each phage lysate was purified and formulated separately via proprietary methods. The purified phage was transferred into APT’s cleanroom for formulation, sterile filtration and aseptic filling of 1 mL into 2 mL single-use vials using a fully enclosed, robotically-operated ISO 5 classified isolator (VanRx Microcell).

**Visual Inspection and Storage.** Finished vials were visually inspected according to USP<790>. All passing vials were labeled and a total of 16 vials were pulled for QC testing for each phage. QC vials and retain stock vials were stored at -80°C or 2-8°C (sterility testing vials). The remaining vials were flash frozen on dry ice and stored at -80°C until shipped.

**Storage and Shipment of the Final Phage Vials.** The final phage vials are stored a glass Schott vial with a Diaikyo cap. All therapeutic phage vials are cryopreserved in -80°C freezers connected to back-up electrical power and actively monitored by an environmental alarm system (Rees Scientific). The therapeutic phage vials are packaged in a vial box and were shipped in a single Styrofoam shipping container with dry ice (< -78°C). The internal temperature of the package was monitored by a temperature monitoring device during transit. Once received at The Johns Hopkins Hospital Pharmacy, pharmacists transferred the vials to an ultra-low freezer (-80°C).

**Potency and Endotoxin**. The potency (phage titer) of the final phage vials were measured via the viral plaque assay method described in APT’s CMC on file with the agency’s under MF 18920. Endotoxin units were quantified using the Limulus Amoebocyte Lysate turbidimetric assay kit (Cape Cod Associates). Methodology was performed per an approved APT Standard Operating Procedure which is based upon manufacturer’s specifications and in observance of USP guidelines. Spike recovery tests were performed to verify suitable test conditions and to verify no inhibition or enhancement of the assay by the cryopreservative glycerol.

**Sterility Testing.** Bacterial sterility testing was performed by plating 100 μL of the filtered product onto a fresh TSA media plate. Test plates and media control plate were incubated at 37°C for 24 hours. No bacterial growth was observed on the test plate or media control plate after 24 hours of incubation. The FDA requested additional sterility testing be performed in accordance to USP <71> testing standards. Sterility testing was performed on a total of 10 final phage vials by a third-party contractor qualified in this methodology.

**Phage Matching for Clinical Case Patient**. Antibacterial activity was measured for PASA16 using the Omnilog System. Three *P. aeruginosa* bacterial clinical isolates obtained were shipped to Adaptive Phage Therapeutics and screened against Pa14NPøPASA16. Pa14NPøPASA16 was introduced to the patient isolate at a MOI of 10 within a 96-well plate loaded with Tetrazolium Dye. The growth of the pathogen in the presence of each phage was measured by aerobic respiration. The results from this screen, showed that Pa14NPøPASA16 was active at least > 44 hours of growth inhibition against the isolates. The major materials used in the manufacturing are listed in APT’s CMC on file with the FDA under MF 18920.
